# Supplementary figures and images for: When Two plus Two Is More than Four: Evidence for a Synergistic Effect of Fatty Acids on Peroxisome Proliferator—Activated Receptor Activity in a Bovine Hepatic Model
Source: Genes (Basel). 2021 Aug 21;12(8):1283. doi: 10.3390/genes12081283 (PMC8393910; doi:10.3390/genes12081283)

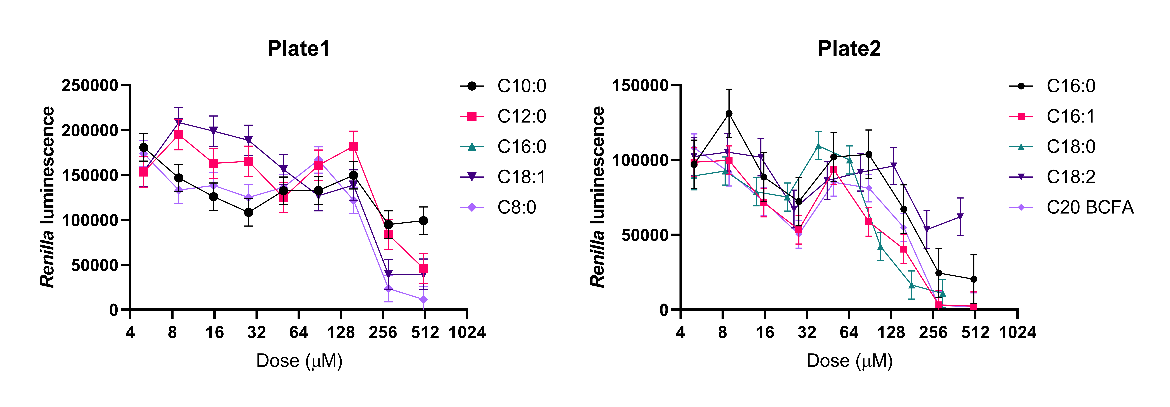

Supplement: Supplementary file 1 [file genes-12-01283-s001.zip › genes-1304300-supplementary.png]
